# Concurrent Anaplastic and Papillary Thyroid Carcinoma Manifesting as a Thyrocutaneous Fistula

**DOI:** 10.7759/cureus.49990

**Published:** 2023-12-05

**Authors:** Syafiqah Razzak Kamel, Khoo Shu Jiun, Kanivannen Arasu, Khairunnisa Muhammad Zuhaidi, Avatar Singh Mohan Singh

**Affiliations:** 1 Otolaryngology - Head and Neck Surgery, Taiping Hospital, Taiping, MYS; 2 Pathology, Taiping Hospital, Taiping, MYS

**Keywords:** papillary thyroid carcinoma, pax8, braf v600e mutations, thyrocutaneous fistula, anaplastic thyroid cancer

## Abstract

Anaplastic thyroid carcinoma (ATC) is a highly aggressive form of thyroid cancer with poor prognosis. Differentiated thyroid carcinoma (DTC) including papillary thyroid carcinoma (PTC) is more common and known to have a favorable outcome after treatment. Here, we report a case of a 59-year-old lady with a long-standing goiter presenting with a discharging anterior neck mass. Fine-needle aspiration cytology (FTAC) of the mass resulted with malignant cells. She underwent surgery, and histopathological examination revealed both ATC and PTC features. Mutation analysis was also performed, and results were positive for BRAF 600VE mutation. She received radiotherapy and also chemotherapy post-surgery. Treatment was well tolerated. The relatively favorable survival of this patient may suggest that synchronous ATC and DTC may have better prognosis than ATC alone. The objective of this article is to report the unique clinical presentation and favorable prognosis with combined treatment modalities.

## Introduction

Anaplastic thyroid carcinoma (ATC) is a type of undifferentiated epithelial thyroid carcinoma. ATC is a rare form of thyroid carcinomas representing only two to three percent of all thyroid gland neoplasms [1]. It is associated with extremely poor prognosis and attributable to more than 50% of deaths related to thyroid neoplasms [2]. Differentiated thyroid carcinoma (DTC), on the other hand, including follicular and papillary subtypes, are more common. Papillary thyroid carcinoma (PTC) alone accounts for 85% of all thyroid carcinomas [3]. Here, we report a case of thyroid carcinoma with concurrent anaplastic and follicular variants of the papillary subtype component presenting as thyrocutaneous fistula with pus discharge.

## Case presentation

A 59-year-old lady with underlying hypertension and a long-standing goiter for 30 years presented to us with increasing size of anterior neck swelling for one month, which was painful with purulent discharge. She denied having shortness of breath, dysphagia, aspiration symptoms, or hoarseness of voice.

Upon examination, the swelling is about 5 cm x 5 cm with erythematous skin, and a punctum was seen anteriorly with pus discharge. Serum T4/TSH at that time was 13.12 pmol/L and 1.34 mIU/L, respectively, which is within normal range. Fine-needle aspiration cytology (FTAC) of the mass yielded malignant cells, which are positive for CKAE1/AE3 and PAX8.

**Figure 1:**
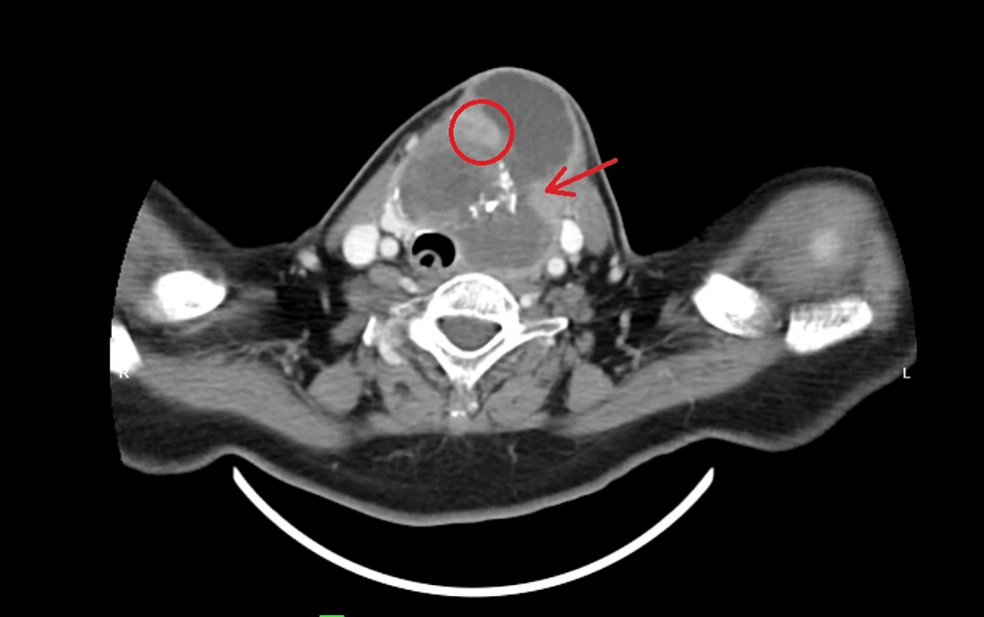
Left thyroid lobe lesion with calcification (red arrow) and solid nodule within (red circle)

CT scan of the neck and thorax revealed a lesion in the left thyroid lobe measuring 6.9 cm x 5.7 cm x 5.9 cm extending into the subcutaneous and skin anteriorly. Within this lesion, there are multiple nodules with calcifications and also solid nodules seen (Figure 1).

**Figure 2:**
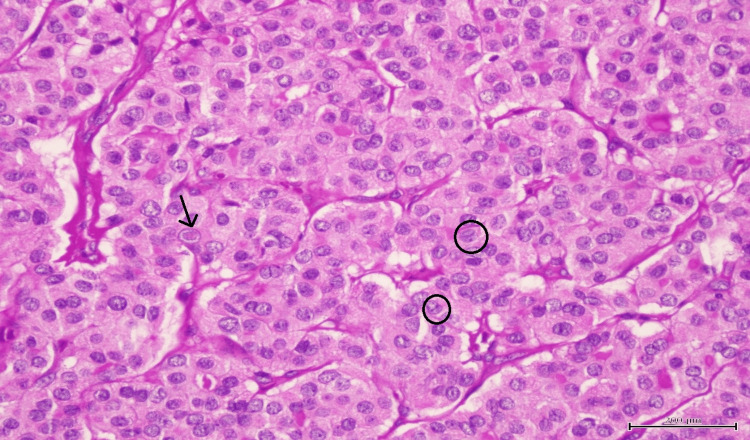
Papillary thyroid carcinoma with nuclear pseudoinclusions (black arrow) and nuclear grooving (black circle).

**Figure 3:**
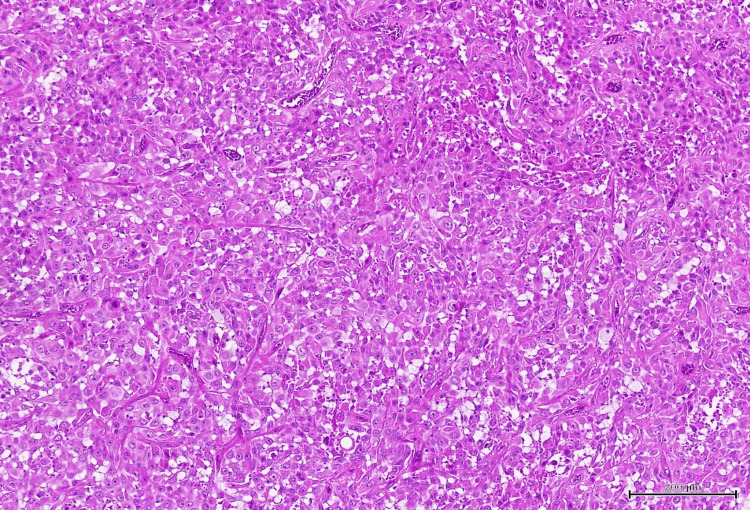
Anaplastic thyroid carcinoma with squamous and epithelioid appearances.

She underwent right hemithyroidectomy and left thyroid tumor debulking surgery and excision of fistulous tract one month after her first presentation to us. Histopathological examination revealed ATC (80%) and follicular variant of papillary thyroid carcinoma (20%) (Figure 2), and the fistula was positive for ATC. The ATC comprises mostly of squamous and epithelioid appearances with small foci showing spindle cell features (Figure 3). Abundant mitoses and foci of tumor necrosis were also seen. Mutation analysis was performed resulting into a positive BRAF V600E mutation.

The patient was referred to another tertiary center, and radiotherapy was initiated followed by chemotherapy afterward.

## Discussion

ATC is the most aggressive form of thyroid malignancy, with studies showing a high mortality rate of 33-50%, a median survival of three to four months, and less than 20% survival of one year [1-4]. Most patients will present with sudden enlarging of neck mass, and no less than 40% of the patients have distant metastases at the time of diagnosis [1]. Similarly, local disease during presentation is often extensive. Most patients complain of local compressive symptoms, such as dysphagia, dysphonia, stridor, and dyspnea. In over 70% of the patients, the tumor infiltrates surrounding tissues, such as fat, trachea, muscle, esophagus, and larynx [5]. The lung is the most common site for distant metastasis, followed by bone [2]. To date, there has been no similar cases reported where the patient presented with a discharging thyroid mass.

Despite the rarity of ATC, cases have been reported in which DTC transformed into ATC and where ATC and DTC occurred simultaneously. The aggressive tall cell variant of PTC is mostly associated with coexistence with ATC. It is a natural course for untreated DTC to develop into ATC [5]. This may be the explanation for the occurrence of both types of carcinoma in our patient given the long-standing history of goiter.

In addition, mutations in proto-oncogenes, such as RAS and BRAF, have been identified in the occurrence of both DTC and ATC. There have been reported cases in which thyroid tumours comprise of both DTC and ATC components histologically and are positive for BRAF V600E mutation, further proving that BRAF mutation is a common driver in dedifferentiation to ATC [5]. This may have occurred in our patient, who has a positive BRAF V600E mutation as well.

Given the rarity and variable disease progression, selecting a therapeutic option is challenging. ATC has a very poor prognosis and responds poorly to conventional therapy. Radioactive iodine also is not efficacious for these patients [6]. According to the American Thyroid Association (ATA), the recommended treatment options for ATC include surgery, radiotherapy, and/or chemotherapy, and these treatment modalities should be combined to improve clinical outcomes [6]. A study done by Haigh et al. showing that the surgical resection of ATC followed by postoperative adjuvant chemotherapy and radiotherapy results in long-term survival [7].

In our patient, the decision for surgical resection was made after reviewing the FNAC result, which yielded malignant cells and was positive for PAX8, which is a transcription factor expressed in a normal and neoplastic thyroid follicular epithelium and only a few other tissues. Its expression is often retained despite transformation into ATC [8]. Moreover, surgical resection was feasible for this patient as the disease did not involve other vital structures in the neck. After the diagnosis of ATC has been made based on the histopathological examination, the patient was subjected for external beam radiotherapy and chemotherapy. The treatment was well tolerated by the patient, and the outcome is yet to be observed once the treatment is complete.

## Conclusions

An unresolved discharging neck mass should alarm clinicians on a more sinister cause rather than just infections. The occurrence of ATC is rare, and it may either coexist with DTC or dedifferentiated from it. We report this case given its rare clinical presentation and favorable prognosis, in the hope that effective treatment regimes can be determined for this fatal thyroid cancer.
